# Supplementary material for: A novel panel of short mononucleotide repeats linked to informative polymorphisms enabling effective high volume low cost discrimination between mismatch repair deficient and proficient tumours
Source: PLoS One. 2018 Aug 29;13(8):e0203052. doi: 10.1371/journal.pone.0203052 (PMC6114912; doi:10.1371/journal.pone.0203052)
Supplement: S1 Table — (DOCX) [file pone.0203052.s001.docx]

**S1 Table: Sample identifiers for whole genome sequences obtained from The Cancer genome Atlas (TCGA) database used in this study.**

| Patient | Tissue Type | SRA Sample Accession Number | Sample ID | Analysis ID |
| --- | --- | --- | --- | --- |
| TCGA-AA-3516 | MSI-H Tumour | SRS130750 | TCGA-AA-3516-01A-02D-1167-02 | 69e9e641-fa2e-4bd9-848e-be5f507660a2 |
| TCGA-AA-3672 | MSI-H Tumour | SRS097008 | TCGA-AA-3672-01A-01D-0957-02 | 9b01e1d4-2cca-49ef-9672-e8692ae621be |
| TCGA-AA-3715 | MSI-H Tumour | SRS097080 | TCGA-AA-3715-01A-01D-0957-02 | 23acfb6f-8071-47cf-9c62-67c22c63e0ec |
| TCGA-AA-3966 | MSI-H Tumour | SRS130791 | TCGA-AA-3966-01A-01D-1109-02 | 4b50f9fa-0fb6-4293-afc5-adc0350f4ed2 |
| TCGA-AA-A00R | MSI-H Tumour | SRS153954 | TCGA-AA-A00R-01A-01D-A077-02 | 300eea0f-bc14-4253-a544-fbc53243ecce |
| TCGA-AA-A01P | MSI-H Tumour | SRS130846 | TCGA-AA-A01P-01A-21D-A079-02 | 60b884be-5009-495f-aec8-bd3be4bf7597 |
| TCGA-AA-A01Q | MSI-H Tumour | SAMN01150758 | TCGA-AA-A01Q-01A-01D-A077-02 | d8e8f805-00c6-467e-a8fc-a2674f1ac38e |
| TCGA-AA-A02R | MSI-H Tumour | SRS154223 | TCGA-AA-A02R-01A-01D-A077-02 | 1d75d53d-94a2-497e-9c4e-f45cf27912d9 |
| TCGA-AZ-4313 | MSI-H Tumour | SRS157354 | TCGA-AZ-4313-01A-01D-1405-02 | a0a2a333-708c-46dd-ab19-c6e7e033d724 |
| TCGA-AZ-4615 | MSI-H Tumour | SRS157387 | TCGA-AZ-4615-01A-01D-1405-02 | ae769553-f8cb-407b-b41a-524adb07282d |
| TCGA-CK-4951 | MSI-H Tumour | SRS159294 | TCGA-CK-4951-01A-01D-1405-02 | ef054dd4-e5ed-4143-80ef-08beffa04d1b |
| TCGA-CM-4746 | MSI-H Tumour | SRS159316 | TCGA-CM-4746-01A-01D-1405-02 | 1878a6ba-0f5c-40b4-a018-7508c8fa3dc2 |
| TCGA-AA-3516 | Matched Normal | SRS130751 | TCGA-AA-3516-10A-01D-1167-02 | e1dbd1cc-89ad-4f93-97f1-982b4ac7f7f3 |
| TCGA-AA-3672 | Matched Normal | SRS097012 | TCGA-AA-3672-10A-01D-0957-02 | 99a5462d-3cb8-464b-98c6-cec13491994c |
| TCGA-AA-3715 | Matched Normal | SRS097084 | TCGA-AA-3715-10A-01D-0957-02 | 6c0543fd-e91d-4cf1-a64b-89ad9e63cf71 |
| TCGA-AA-3966 | Matched Normal | SRS130801 | TCGA-AA-3966-10A-01D-1109-02 | 078906fa-6e88-4626-b01f-9b529b969460 |
| TCGA-AA-A01P | Matched Normal | SRS130854 | TCGA-AA-A01P-11A-11D-A079-02 | 74847765-b70c-47c8-9eb4-d5eae2e4c704 |
| TCGA-AA-A01Q | Matched Normal | SAMN01152602 | TCGA-AA-A01Q-10A-01D-A078-02 | 0e3b9a0b-8fd8-4726-bcd8-d5b8563bb630 |
| TCGA-AA-A02R | Matched Normal | SAMN00152611 | TCGA-AA-A02R-10A-01D-A078-02 | 0f21aa03-df30-4b29-b908-daf9584088d6 |
| TCGA-AZ-4313 | Matched Normal | SRS157361 | TCGA-AZ-4313-10A-01D-1405-02 | 7c0a3b4d-0fc0-4b9a-b4c4-d075b8117f42 |
| TCGA-AZ-4615 | Matched Normal | SRS157394 | TCGA-AZ-4615-10A-01D-1405-02 | af33c9f9-02a6-4e4a-9f1d-52f04bfa6116 |
| TCGA-CK-4951 | Matched Normal | SRS159301 | TCGA-CK-4951-10A-01D-1405-02 | 5ac4252e-6cb6-4226-a8cd-489a9986c61e |
| TCGA-CM-4746 | Matched Normal | SRS159323 | TCGA-CM-4746-10A-01D-1405-02 | a9540af8-5f10-4d06-a31d-1e25a69e31bd |
| TCGA-AA-3509 | MSS Tumour | SRS156892 | TCGA-AA-3509-01A-01D-1405-02 | 4e552949-246f-4788-a760-9b6a23d89bf3 |
| TCGA-AA-3555 | MSS Tumour | SRS196934 | TCGA-AA-3555-01A-01D-1637-02 | 875b31fd-9e8a-49d2-89b4-d8256f89ef5a |
| TCGA-AA-3558 | MSS Tumour | SRS130763 | TCGA-AA-3558-01A-01D-1167-02 | 1b21ee51-605f-478b-8a82-e25a3fa9b678 |
| TCGA-AA-3685 | MSS Tumour | SRS130776 | TCGA-AA-3685-01A-02D-1167-02 | 25f4344f-ea46-48ac-b2a9-74f9222fb8aa |
| TCGA-AA-3693 | MSS Tumour | SRS097064 | TCGA-AA-3693-01A-01D-0957-02 | 3a9f1142-0d5b-4583-a570-4da8e1455e0c |
| TCGA-AA-3968 | MSS Tumour | SRS130808 | TCGA-AA-3968-01A-01D-1167-02 | 3f6440b7-1298-4892-a133-3d48eb885eda |
| TCGA-AA-3970 | MSS Tumour | SRS130814 | TCGA-AA-3970-01A-01D-1109-02 | d67dcadf-7893-42fd-afd7-e2ed9a1aa33d |
| TCGA-AA-A00U | MSS Tumour | SRS153966 | TCGA-AA-A00U-01A-01D-A077-02 | 285ce8fc-dc1b-4188-bb8e-723573a9545a |
| TCGA-AY-4070 | MSS Tumour | SRS133582 | TCGA-AY-4070-01A-01D-1109-02 | acec5f0d-3fa8-45ca-bdda-3058c14bbcc0 |
| TCGA-AY-4071 | MSS Tumour | SRS133599 | TCGA-AY-4071-01A-01D-1109-02 | 1b3451c6-b020-4a04-b454-018ceb86da2f |
| TCGA-CA-5256 | MSS Tumour | SRS159111 | TCGA-CA-5256-01A-01D-1405-02 | 1cad444d-0ed8-437d-8f26-66e103379160 |
| TCGA-CM-4748 | MSS Tumour | SRS159338 | TCGA-CM-4748-01A-01D-1405-02 | bc5d8cfa-f666-4b3a-9117-5b60f92d480e |

Access identifier: phs000178.v8.p7 DAR: 17798

Request date: 2012-11-13

Study accession: phs000544.v1.p6

Parent study: phs000178.v7.p6
