# Supplementary material for: A novel panel of short mononucleotide repeats linked to informative polymorphisms enabling effective high volume low cost discrimination between mismatch repair deficient and proficient tumours
Source: PLoS One. 2018 Aug 29;13(8):e0203052. doi: 10.1371/journal.pone.0203052 (PMC6114912; doi:10.1371/journal.pone.0203052)
Supplement: S2 Table — (DOC) [file pone.0203052.s002.doc]

**S2 Table: List containing 300-500bp amplicon or repeat name of 120 markers, amplicon position (hg19), PCR primers sequences, SNPs in close proximity to mononucleotide repeats and AUC of 41 selected markers.**

| **Identifier ^a^** | **Repeat length (bp)** | **Repeat motif** | **Amplicon position** | **Primer** | **SNPs scored  for allelic bias** | **AUC ^b^**  **(41 markers)** |
| --- | --- | --- | --- | --- | --- | --- |
| GM01 | 10 | A | chr11:28894282-28894553 | 5' TCAAGGCCAGGCAATTAATCAG 3' 5' ACTTGCTGAATGTCCAAGGTG 3' | rs7951012 | 0.94 |
| GM02 | 11 | A | chr1:116245990-116246244 | 5' GTGCTACATGAGATAGCTGGGA 3' 5' CTCTTCTGGCCAGTTCTATGTGT 3' | rs10802173 rs148789685 |  |
| GM03 | 8 | A | chr4:120206298-120206557 | 5' TGGAGTAAGACCCTTTAGGCAG 3' 5' AGACTCTGGAAGCAAATGGCA 3' | rs17050454 rs10032299 |  |
| GM04 | 7 | A | chr13:92677409-92677684 | 5' CCTTTTGGCCAGAATATGCC 3' 5' GGCATGAGGAAGTGAAGGGA 3' | rs9560900 |  |
| GM05 | 9 | A | chr2:216770642-216770900 | 5' AGGTGTCAAGCAAGGACTCAG 3' 5' AGGCGTTTTCACGTTGGAGG 3' | rs6704859 |  |
| GM06 | 9 | A | chr16:77496387-77496667 | 5' AGAGGCAGAATGTGGAAAAGTC 3' 5' GCATTCTCCCACAGCACAAT 3' | rs6564444 rs143453795 rs145573459 |  |
| GM07 | 11 | A | chr7:93085548-93085828 | 5' GGAGGGACATGTGTTTCCAAAT 3' 5' CACAATGAGCCAAGTCTCACA 3' | rs2283006 | 0.97 |
| GM08 | 8 | A | chr21:36574923-36575189 | 5' AGCAACCTCTTAAATCCAGTACT 3' 5' TGGGCTTTCTTGACTTTGGA 3' | rs2834837 rs115025058 |  |
| GM09 | 8 | A | chr20:6836843-6837099 | 5' TTTCTCAGGACAAAGAGCAAGGT 3' 5' CTGGGTTCCATCTTGTGGGG 3' | rs6038623 | 0.82 |
| GM10 | 9 | A | chr1:59891529-59891795 | 5' ATCAGCTGACTCCTTACCCT 3' 5' TGGGGTGAGAGATGGACATG 3' | rs946576 rs182557762 |  |
| GM11 | 9 | A | chr5:166099809-166100081 | 5' CTCATGGTTAATACAATTAGGCACA 3' 5' ACATGGTGTGCTACCTTTCA 3' | rs347435 | 0.93 |
| GM13 | 11 | A | chr12:107492450-107492711 | 5' TTCTTCAGGGCCCATTATTGT 3' 5' TGAGGAATGTGCAGTTGACAC 3' | rs34040859 rs77265275 rs201488736 |  |
| GM14 | 11 | A | chr3:177328721-177329014 | 5' AGCTTGGCCATATTTGTGCA 3' 5' ACTTGATAGGGTTAAATGTCCGT 3' | rs6804861 | 0.87 |
| GM15 | 9 | A | chr7:97963570-97963830 | 5' TGCCTTCGAGTTTAAATGCCT 3' 5' GCCTCGTTATTTTGTGTGCC 3' | rs6465672 | 0.87 |
| GM16 | 8 | A | chr6:100743524-100743782 | 5' GCCACACTGACTTTGAACCTT 3' 5' ACAGCTTCTTCCTCACTCTACT 3' | rs7765823 |  |
| GM17 | 9 | A | chr11:95550977-95551231 | 5' TCCCTAGAAAGAGAACGACAACA 3' 5' AAATGCCCACCAAGATTGTAAAA 3' | rs666398 | 0.88 |
| GM18 | 12 | A | chr10:8269462-8269727 | 5' GGGGAGAAGACGGTTGAACT 3' 5' ACTGGTTCACTGGCCTTTTG 3' | rs113251670 rs189036006 rs533236 |  |
| GM19 | 7 | A | chr11:114704247-114704523 | 5' AGGTAAAGTCAGACACAATCCCA 3' 5' ACCCTCATGTTTCCCACCTCA 3' | rs142833335 rs190597109 rs10502196 |  |
| GM20 | 8 | A | chr7:142597420-142597679 | 5' GCAATCACATTTGCATTGGTTTT 3' 5' TGACTATGAGCTCCACAAACGTA 3' | rs6961869 rs6961877 |  |
| GM21 | 9 | A | chr3:142695286-142695560 | 5' TTCTCCATTGGAAGTATTTGGGA 3' 5' TGTGTATTCAGGGTCCAGGG 3' | rs185182 | 0.53 |
| GM22 | 10 | A | chr14:43400950-43401207 | 5' TCATAACCAAGAGCACCACCT 3' 5' TGTGATAGGGAAACACACGGA 3' | rs58274313 | 0.97 |
| GM23 | 9 | A | chr5:11345800-11346075 | 5' CAGCATAAATCCAATGGCTATG 3' 5' TCAGATTGCAAAGGGGTACA 3' | rs184237728 rs32123 | 0.68 |
| GM24 | 7 | A | chr10:117432031-117432299 | 5' AAACATTTCGACTGGTGCAA 3' 5' TTCTTCTTTCCCCCAAATGA 3' | rs2532728 |  |
| GM25 | 7 | A | chr3:110871894-110872161 | 5' TGGGATTAGGGAAGGGAGAG 3' 5' GGCCCTCCCCAACTAAAAT 3' | rs74593281 rs6437953 rs188039266 |  |
| GM26 | 10 | A | chr14:49584656-49584913 | 5' CCTTCCTTTGATCCGCAAGC 3' 5' CTGCCACCTAGGAACTGGAG 3' | rs187027795 rs11628435 | 0.87 |
| GM27 | 7 | A | chr11:85762061-85762349 | 5' TTTTTGTTGCCCATTTCCTC 3' 5' AGGGTACTGACCCTAGCTCCA 3' | rs669813 rs181565251 rs146406522 |  |
| GM28 | 9 | A | chr5:29209275-29209526 | 5' CTCAGACAAAGACATACGAAGCC 3' 5' TTGGTTCTACAGTAATTGTGCTTCT 3' | rs4130799 | 0.54 |
| GM29 | 10 | A | chr3:70905468-70905731 | 5' CCCTCCCAAATGTCAAGTGT 3' 5' CCCACCCACACTCTTTTGTT 3' | rs2687195 | 0.88 |
| GM30 | 7 | A | chr14:53111446-53111710 | 5' TCAATGCTATTGGCCTATAAAGAGT 3' 5' ATGCATTTCCTTCTGGCCTA 3' | rs12880534 |  |
| IM07 | 10 | A | chr6:100701756-100702050 | 5' TCACCATCATCACCATGCTT 3' 5' TCTGGCAAACTCTTCACTGG 3' | rs189035042 rs6915780 |  |
| IM12 | 10 | A | chr8:23602751-23603036 | 5' AGTGGAGAAAACGGTTGTGG 3' 5' GAAGGCAGACAAGGGATTCA 3' | rs389212 |  |
| IM13 | 7 | A | chr2:235496873-235497180 | 5' GTGACCGCACAAAGTCACAC 3' 5' TCCAACAATCACAGTCCATGA 3' | rs6721256 rs183025093 rs187312036 |  |
| IM14 | 7 | A | chr7:80104285-80104624 | 5' TCAAGACTCAGCCATTTCCA 3' 5' GGAAGCTGAGAGCAGGTTTTT 3' | rs11760281 | 0.74 |
| IM15 | 8 | A | chr6:91455016-91455307 | 5' TCGTCAGGCTCTGCAACTAC 3' 5' CGATGGGATTGAATTTGGAT 3' | rs1231482 |  |
| IM16 | 9 | A | chr18:1108609-1108894 | 5' AGGACCTCGAGCTTCTCTTT 3' 5' TTCTTTTGCTTCCGTGTGTG 3' | rs114923415 rs73367791 rs59912715 | 0.97 |
| IM17 | 9 | A | chr13:31831349-31831705 | 5' TGCAACCAGAGGTTTTAATCG 3' 5' CTCAATTCAGCAACAGGTCA 3' | rs932749 |  |
| IM19 | 7 | A | chr9:82474924-82475277 | 5' CAACCACAGTTTGCCAGCTA 3' 5' TCCTTGCTATCATTTGGAGAGA 3' | rs72736428 rs186539440 rs4877153 | 0.65 |
| IM20 | 7 | A | chr13:57644542-57644833 | 5' CCAGTTTCACATTTCGCTTGT 3' 5' TGGCAACAAAACAGTAACAGGA 3' | rs6561918 |  |
| IM21 | 8 | A | chr1:215136329-215136605 | 5' AGTGAATGGGCTTTGGACTG 3' 5' AACTGGAGTGGGTGAACCTG 3' | rs181787229 rs1901621 rs1901620 |  |
| IM22 | 7 | A | chr7:90135380-90135698 | 5' CACCAGCTTTTCTCCCTTCA 3' 5' TGGCACTCAATACCAAACTGG 3' | rs557365 |  |
| IM23 | 7 | A | chr6:72729441-72729714 | 5' GGTTTCTGTGCTGAATCTTGG 3' 5' AACCCCAGTTTTCTGCCTCT 3' | rs10771087 |  |
| IM25 | 8 | A | chr12:24568297-24568575 | 5' CCATGGTACCACTGTGGAGT 3' 5' TAGAGGGGGCTTGAATGTTG 3' | rs2863375 |  |
| IM26 | 7 | A | chr3:166053374-166053712 | 5' GGGCTCGACTTGATTTACGA 3' 5' GGGAAGCAATCTCATGGCTA 3' | rs4723393 rs112516918 |  |
| IM27 | 7 | A | chr7:35079029-35079302 | 5' ACGCATGGAAAAAGAGGTTC 3' 5' CAAGGCTGGTATGGGTCAAT 3' | rs10815163 |  |
| IM28 | 11 | A | chr9:5122829-5123102 | 5' TGTGGAATCCCTCCTGAAAT 3' 5' CCGCTGGTGGACTTTTACTC 3' | rs8087346 |  |
| IM32 | 11 | A | chr18:42045361-42045640 | 5' GCCAAAATGCCTAACTCCAA 3' 5' GGACTCGGATGGAAGACAAA 3' | rs202225742 rs35644463 rs113180202 |  |
| IM33 | 10 | A | chr8:25731833-25732120 | 5' AGGGTATGATTTGGGGGTGT 3' 5' GTGGACCAAAGGAGCAGAAG 3' | rs1524881 |  |
| IM34 | 10 | A | chr7:83714549-83714816 | 5' TGAGGGTGGATGCTTCATTT 3' 5' CAGGATATTCCTCAGTTCAGTTCC 3' | rs67283158 rs10792775 rs116387070 |  |
| IM35 | 10 | A | chr11:84425027-84425322 | 5' TCAAATGCAGACTCAACATGA 3' 5' AGCAGAGGAGCCATCAATTC 3' | rs2331498 |  |
| IM37 | 10 | A | chr17:50813421-50813720 | 5' CAGGCACACACACTTTCGTT 3' 5' TTCTCATGCAGTCAACCATTG 3' | rs76771828 rs190979688 rs187315716 |  |
| IM39 | 8 | A | chr2:103233602-103233932 | 5' AGACGTCCAAAGGTCGCTAA 3' 5' CCCTCACTGCCTGTAAACCT 3' | rs10516683 |  |
| IM40 | 8 | A | chr4:84074695-84074985 | 5' ATCACAAAAACAGGGGCCTA 3' 5' CCTTGTCTGGCTCAATCACC 3' | rs1944640 rs112075239 |  |
| IM41 | 8 | A | chr6:147948700-147949027 | 5' CTGCTCCACATTCCCATTCT 3' 5' TGGCAGGAAACATCTGTTCA 3' | rs1409192 | 0.61 |
| IM42 | 9 | A | chrX:96502491-96502781 | 5' TGGCTGAGTAAAATGGTGACA 3' 5' GCTTGGGGGAATTTCTTGAT 3' | rs9981507 |  |
| IM43 | 7 | A | chr21:32873526-32873866 | 5' CAGAAGGTCAGGACCACACA 3' 5' ATTTGGTGGGTTCCAGTGAG 3' | rs201750704 rs4763716 | 0.65 |
| IM44 | 9 | A | chr12:9796844-9797182 | 5' CCTCCTAGCATTCCATAGCAC 3' 5' TGCAACCTCGTAAGCTCATTT 3' | rs189419054 rs2178216 |  |
| IM45 | 11 | A | chr4:99545274-99545564 | 5' GCCACATTTGCTGGTATTCA 3' 5' TTTTTCCTCTGGGAAACCAT 3' | rs2588655 rs149325240 rs232496 |  |
| IM47 | 12 | A | chr21:22734257-22734517 | 5' TGGTTCAGACATACACGTACAGG 3' 5' ATAACAGGCACAAGGGTGGA 3' | rs7642389 |  |
| IM49 | 12 | A | chr3:56681883-56682149 | 5' CCTGGCAAATGATGCTTTAGA 3' 5' CCTCCCTCCTAGGCTCAAGT 3' | rs1739651 rs145870165 | 0.96 |
| IM50 | 12 | A | chr20:37047920-37048224 | 5' CGAGGCGGGTATTTACTTGA 3' 5' GGAGTTGGGGCAAAAATCAC 3' | rs4836397 |  |
| IM51 | 12 | A | chr5:128096936-128097255 | 5' CAAACCCCCGAGACACAC 3' 5' AACGTGGCTCTTTATCCCATT 3' | rs74462385 rs9982933 rs2155801 |  |
| IM52 | 11 | A | chr21:22846659-22846944 | 5' GATGGAGGGCCCTTTAATTT 3' 5' CGATGAAGTGGTTGATGTGAG 3' | rs182630429 rs140426089 rs12352933 |  |
| IM53 | 11 | A | chr9:20662482-20662766 | 5' GACAACTCCGAAGGGCAATA 3' 5' AGTTTGGGTTGCAAGACGTT 3' | rs13046776 |  |
| IM54 | 11 | A | chr21:33709922-33710213 | 5' GCAACATTGAAATGCTGGAA 3' 5' TAACATTTGGGAGGGGGAAT 3' | rs13099818 |  |
| IM55 | 7 | A | chr3:143253627-143253930 | 5' GCTGAATAGCGGGATCAAAA 3' 5' GGAATTAGGTACCAGATCTCCTTT 3' | rs35085583 | 0.53 |
| IM57 | 8 | A | chr3:81209863-81210156 | 5' GATTATCAGCCCAGGGAGGT 3' 5' ATGGCAGCACTGGGAAATTA 3' | rs10156232 |  |
| IM59 | 8 | A | chr8:108358809-108359137 | 5' TATGGCTGCAGCATTACCAG 3' 5' GCCAGAGTCCACAGACTCAA 3' | rs34696106 | 0.70 |
| IM61 | 7 | A | chr12:73576301-73576606 | 5' GAGCAAGGCATTTGAATCTG 3' 5' ATATGAGGCGCTCTCTCTCG 3' | rs34764455 |  |
| IM63 | 8 | A | chr3:115815913-115816216 | 5' TGCCTTTGGTTGTACCTTTG 3' 5' TCAAGTGAGCCTTGTGGAAA 3' | rs201451896 rs112858435 rs75477279 |  |
| IM64 | 12 | A | chr16:14215981-14216240 | 5' CCTTCCCCGTTCTTTCTCTT 3' 5' AAGGTAGGTGACCGGCTGAT 3' | rs7324645 rs9511253 |  |
| IM65 | 11 | A | chr13:25000797-25001149 | 5' GCATCTCAAACTGTGCCTGT 3' 5' CACGGGTCTAACTGTCCTCA 3' | rs147847688 rs141474571 rs4794136 |  |
| IM66 | 7 | C | chr17:48433883-48434148 | 5' CCACTCCAGCAAGTCTCCAG 3' 5' CAAGGGCCTGCTGTATGTCA 3' | rs67082587 rs57484333 | 0.79 |
| IM67 | 7 | C | chr7:22290637-22290990 | 5' AGCCCATGTTTTCCACAGAA 3' 5' TACCAGGTGCCCTAAACAGG 3' | rs557365 | 0.79 |
| IM68 | 8 | C | chr12:129289515-129289789 | 5' TTCTAGACACAGACGCACACG 3' 5' GGGACTGCCACTAGTAGCTCA 3' | rs10847692 |  |
| IM69 | 7 | C | chr9:92765658-92765989 | 5' TGGGGGCAGTTTCTATTCTG 3' 5' ATCAGTTTTCGATGGGGAGA 3' | rs1036699 |  |
| LR01 | 11 | A | chr13:97387292-97387567 | 5' TTGGATGCTGGATTTTGACA 3' 5' CTCATATCCCCCTCCCAGAA 3' | rs1924584 rs4771258 |  |
| LR02 | 8 | C | chr4:134947615-134947875 | 5' TATTGGCCAGGAATTTTTGC 3' 5' GGAGCTCACGCTAATGACCT 3' | rs189671825 rs192703656 rs1494978 |  |
| LR04 | 7 | C | chr1:4676948-4677234 | 5' CCCCAAGCTGTTTCCTCCAT 3' 5' GCTGGGGCAAGAAATTCAGC 3' | rs113646106 rs2411887 |  |
| LR05 | 9 | C | chr2:10526489-10526814 | 5' GAGCTGCCTACTCGCTGACT 3' 5' GCCACTGATGACAACCTCCT 3' | rs111286197 rs13431202 |  |
| LR06 | 7 | C | chr18:20089314-20089588 | 5' CATCTAGCATTCTCTCATTTCAGC 3' 5' TGCCAAAACCAAAGACAAGG 3' | rs501714 |  |
| LR08 | 7 | C | chr11:56546008-56546315 | 5' GGCTGCTTAAGGGAAAGTGC 3' 5' CGTGTTTTGGTCAAAGTTGTG 3' | rs181578273 rs7117269 | 0.57 |
| LR10 | 9 | A | chr1:81591297-81591555 | 5' ATGTTTGGTGCATGAAATCTG 3' 5' TGAGTTCCACATGGCTCTTG 3' | rs111814302 rs1768398 rs1768397 | 0.84 |
| LR11 | 11 | A | chr2:217217726-217218005 | 5' TATTCCCCTTGTGTGGGAGA 3' 5' CAAAGAGAATGGGTGGGAGT 3' | rs13011054 rs147392736 rs139675841 | 0.92 |
| LR12 | 11 | A | chr14:47404086-47404346 | 5' GGTGAGGAAAGCACAAGGTC 3' 5' CCGTGGAATTTCTTCTGCAC 3' | rs187434561 rs144159314 |  |
| LR13 | 7 | A | chr8:21786845-21787107 | 5' TCCTCGTCCTCTCAGATGTGT 3' 5' TCAGGACTTAGCACCAGGAAA 3' | rs2127206 |  |
| LR14 | 9 | A | chr17:69328365-69328640 | 5' CCCGTTTTCAGACCAAGTGT 3' 5' TTGGAACAGGATGGGTGAAT 3' | rs9895642 |  |
| LR15 | 7 | A | chr8:92077118-92077383 | 5' TGATTCGGGCTTGGACTTAG 3' 5' GTCAATCACTTTGCCTGCTC 3' | rs56084507 | 0.69 |
| LR16 | 11 | A | chr3:8522305-8522590 | 5' GTTTGATCTCTGGCCCTGTC 3' 5' GCCTCCTTAATCTCCTCCATC 3' | rs148171413 rs6770049 |  |
| LR17 | 11 | A | chr14:55602913-55603194 | 5' AGACCACCCCTTAGGCAAAC 3' 5' AGTGCAGCAAGGCAGATGAG 3' | rs79618905 rs77482253 rs1009977 | 0.98 |
| LR18 | 8 | A | chr1:220493800-220494106 | 5' TGGGGAGGGAACCTCATTAC 3' 5' CAGTGCCTGTTGAGTAGAACC 3' | rs191265856 rs199830128 rs74940412 |  |
| LR19 | 8 | A | chr12:29508532-29508843 | 5' TGAGTGCTGCTCATATTTTTCC 3' 5' GGGGCTTCAGTCTCAGGATAG 3' | rs10843391 rs186762840 |  |
| LR20 | 8 | A | chr1:64029521-64029836 | 5' TCAGCCTATGAAGATCCTCTG 3' 5' AAGGAAGACGGGGAAGACTG 3' | rs146973215 rs191572633 rs217474 | 0.91 |
| LR21 | 9 | A | chr15:50189339-50189607 | 5' TGGGTACAAAGCTCAAGTCAAC 3' 5' TCTCCAAAGGCTTCTCCTTG 3' | rs182900605 rs80237898 rs2413976 | 0.83 |
| LR23 | 11 | A | chr2:142013847-142014151 | 5' TGTAGCCTAGGTAAAGAGGACAA 3' 5' CATTTAGCATTTTGCCATTCC 3' | rs434276 rs146141768 |  |
| LR24 | 9 | A | chr1:153779290-153779565 | 5' TATGCCTTCTGGAGGAGTGG 3' 5' TGGAATAGCGGTAAGGCTTG 3' | rs192329538 rs1127091 | 0.91 |
| LR25 | 7 | A | chr16:63209414-63209676 | 5' TTAACCTGCCAGCTCAGTTC 3' 5' GCTTCCACTCATTTGCATTG 3' | rs76192782 rs79880398 rs4949112 |  |
| LR26 | 10 | A | chr16:80050164-80050433 | 5' TGCATAGGCAGACCTCAAAAC 3' 5' GAAAGCCTGATGTTTGACACC 3' | rs4889066 rs187883346 |  |
| LR27 | 8 | A | chr4:72877320-72877604 | 5' TTTGGTCATTGCTGTCATGG 3' 5' CAACAAGGAATTGAATGATGC 3' | rs55894427 rs74733006 |  |
| LR28 | 9 | A | chr12:81229619-81229925 | 5' TGAGTCCCTTTTGAAATGTTG 3' 5' GCCAACCAATGGAGTTTTAAG 3' | rs185642078 rs28576612 rs10862196 |  |
| LR29 | 10 | A | chr6:78198189-78198498 | 5' CAATGTTTGATTAACCATGACG 3' 5' GCACTTTTCTCACACAATTTGG 3' | rs1778257 |  |
| LR30 | 10 | A | chr11:105444906-105445201 | 5' GCAGGAATTCATTCTGAAGC 3' 5' AACGCAGTGAGGAACAAAGG 3' | rs7933640 |  |
| LR31 | 8 | A | chr3:62995387-62995657 | 5' TGGATTTGCATCTGTGAATTG 3' 5' TTTTGATGGCTTTTACTTTTCC 3' | rs183248146 rs2367592 |  |
| LR32 | 10 | A | chr19:37967035-37967313 | 5' CTGCCTATGCCAAACAAATG 3' 5' AGCACAAGCCTTTTGTCAGC 3' | rs7253091 | 0.91 |
| LR33 | 11 | A | chr4:138498516-138498782 | 5' GAATAGCGGGAAGAACTGGA 3' 5' TGCATTCGAATCAGGAATGA 3' | rs200714826 rs4637454 rs111688169 |  |
| LR34 | 9 | A | chr3:115376990-115377261 | 5' CCCATCCTTAGACCCCAGAC 3' 5' GAAAATGAGACGCGAAAAGG 3' | rs187521190 rs192106258 rs9883515 |  |
| LR35 | 10 | A | chr8:130384312-130384584 | 5' AAAGCTTGTGGGTGATGGAG 3' 5' TGCTTGGAATAGGATGCTTTG 3' | rs4733547 |  |
| LR36 | 12 | A | chr4:98999555-98999845 | 5' TCCCCAGGACCCTAGTCTTC 3' 5' GGTGGCAAGCACTTTTGTAAG 3' | rs182020262 rs17550217 | 1.00 |
| LR39 | 10 | A | chr17:66449171-66449485 | 5' AGCATGGGAATAACGACAGG 3' 5' TCGTTGTGTTGGAGGTAGAGC 3' | rs2302784 |  |
| LR40 | 9 | A | chr2:13447304-13447570 | 5' AAATGAACACTATGCATGTCAGG 3' 5' TTGCCTCTTGCAACTGATTG 3' | rs6432372 | 0.90 |
| LR41 | 12 | A | chr4:34073929-34074197 | 5' CATGGACCGCTGATCTCTG 3' 5' GGAGGGATCTAGCCACCAC 3' | rs190518698 rs6852667 |  |
| LR43 | 12 | A | chr5:86198899-86199207 | 5' GGCAACAGCCTCATAACTGC 3' 5' GCTGTCTCCTGGCTCTAACC 3' | rs201282399 rs10051666 rs6881561 | 0.98 |
| LR44 | 12 | A | chr10:99898182-99898454 | 5' TTTGGCTGGGCCTGGTAG 3' 5' CAGAGTGCACCTCAGTGACC 3' | rs78876983 rs7905388 rs7905384 | 0.99 |
| LR45 | 7 | A | chr2:226937965-226938246 | 5' TGCAGAGAAGAGATACAGAAAGC 3' 5' TGCAAAAATCCCAGATTGAAG 3' | rs180896305 rs1522818 rs144175764 |  |
| LR46 | 8 | A | chr20:10659968-10660261 | 5' GAGTGTGGGAGAAGTCCTACG 3' 5' TTCAGGAGATGAAAAGGCTTG 3' | rs143884078 rs182346625 rs6040079 | 0.90 |
| LR47 | 7 | A | chr10:20506574-20506830 | 5' TCCCTGAAGGAAGGAAAAATC 3' 5' GTGATTGTGAAGTTGGATTTGC 3' | rs11597326 rs12256106 |  |
| LR48 | 11 | A | chr12:77988002-77988288 | 5' ATTACCCATGGGGGATGTTG 3' 5' AGTTGGGGAACATTCCTTCC 3' | rs11105832 | 0.99 |
| LR49^c^ | 7 | A | chr15:93618885-93619163 | 5' ATCTGTAAGGATCGGGCTGA 3' 5' CAACACAACGCCATACTGCT 3' | rs80323298 rs201097746 rs12903384 | 0.95 |
| LR50 | 7 | A | chr2:76556173-76556470 | 5' TTCCCCATTTGATGATCCTG 3' 5' AGAGTTTTCCCCACTCAGCA 3' | rs925991 rs144630203 |  |
| LR51 | 7 | A | chr10:51026570-51026831 | 5' TGAATATGCCTCAAGCACCA 3' 5' AATGCAAACCTCCTAGGTTAAAA 3' | rs8474 | 0.69 |
| LR52 | 12 | A | chr16:63861273-63861586 | 5' GTGCTCTGCATCTCATACGC 3' 5' CCTCCTTGGCTAACTTGCTC 3' | rs2434849 | 0.88 |

^a^ Use to designate the mononucleotide repeat and the amplicon.

^b^ Determined for selected markers as described in the text.

^c^ For LR49 only the length of the poly-adenine tract was scored. This tract is adjacent poly-guanine tract of length seven.

AUC: Area under the curve
