# Supplementary material for: A novel panel of short mononucleotide repeats linked to informative polymorphisms enabling effective high volume low cost discrimination between mismatch repair deficient and proficient tumours
Source: PLoS One. 2018 Aug 29;13(8):e0203052. doi: 10.1371/journal.pone.0203052 (PMC6114912; doi:10.1371/journal.pone.0203052)
Supplement: S3 Table — (DOCX) [file pone.0203052.s003.docx]

**S3 Table: List containing amplicon or repeat name (100-150bp) of 17 marker panel, amplicon position (genome build hg19), PCR primers sequences and SNPs in close proximity to mononucleotide repeats.**

| **Identifier ^a^** | **Repeat**  **length (bp)** | **Repeat motif** | **Amplicon position** | **Primers** | **SNPs scored  for allelic bias** |
| --- | --- | --- | --- | --- | --- |
| AP003532_2 | 9 | A | chr11:127624984-127625150 | 5' ACTGTGGTTTTAATTTGCATTTCCC 3' 5' TGTGCCTTTAAAGTGACCTT 3' | rs10893736 |
| DEPDC2 | 8 | C | chr8:68926646-68926777 | 5' GTTCACACACATGCAAGCTG 3' 5' GAAGGGTAGGGAGATGCAGA 3' | rs4610727 |
| GM07 | 11 | A | chr7:93085649-93085797 | 5' GGTGGCTTGTTTTCATTTTGTC 3' 5' CATATGGGGTTTGGTCACATTTT 3' | rs2283006 |
| GM09 | 8 | A | chr20:6836938-6837051 | 5' TCCGTATTCCAGGAGTAAGAGT 3' 5' CTCAGAGGGAAGGTGGCA 3' | rs6038623 |
| GM11 | 9 | A | chr5:166099845-166099965 | 5' CCTACGTATCTAAGTATTCTCCAGC 3' 5' ACAGTGGGTTTCAAATGTCACTTC 3' | rs347435 |
| GM14 | 11 | A | chr3:177328763-177328864 | 5' CCCAGGCTAAAAGACCAAGA 3' 5' CAGCAAAGGATAAACATTGTGGA 3' | rs6804861 |
| GM17 | 9 | A | chr11:95551064-95551249 | 5' AGAAGTCAGTGCATGTGTCTT 3' 5' CCCACCAAGATTGTAAAATGTGA 3' | rs666398; rs528485045 |
| IM16 | 9 | A | chr18:1108732-1108867 | 5' GAATCAGCAGTGTTCATACCTTC 3' 5' TTGTTCACTTTAGTAGGAACTGGT 3' | rs73367791 |
| IM49 | 12 | A | chr3:56681995-56682136 | 5' GGTAGTTGGATCGCTTCAGG 3' 5' CAGCCTCTTGAGTAGCTTGG 3' | rs7642389 |
| IM66 | 7 | C | chr17:48433923-48434025 | 5' CAGGAGGTGCTGGAAATCC 3' 5' CATCAGCCGCGTCGTAGG 3' | rs4794136 |
| LR11 | 11 | A | chr2:217217787-217217903 | 5' TCCTGTGGTCTGTGAAGCTA 3' 5' GTGCATTTGAACATCGCCTC 3' | rs13011054 |
| LR20 | 8 | A | chr1:64029549-64029704 | 5' GGCATTGCCCCTATATACTGT 3' 5' TTCCCAGTTCTGAATCTAGAAAGA 3' | rs146973215; rs217474 |
| LR24 | 9 | A | chr1:153779392-153779496 | 5' GGTAACCAAAGCAGGAAAACATT 3' 5' CCCTCTCTCCCTGGAATAAGT 3' | rs1127091 |
| LR36 | 12 | A | chr4:98999679-98999817 | 5' GTGGTGACCCTGAACGTTAA 3' 5' CCTGGGTGTAAATGATGGGAA 3' | rs17550217 |
| LR44 | 12 | A | chr10:99898232-99898370 | 5' GAGGCCAAGAGTTCAAGACCA 3' 5' GATGAGAATTAGCATACCTTCCA 3' | rs7905384; rs7905388 |
| LR48 | 11 | A | chr12:77988077-77988147 | 5' GGGAGGAAGTATCTGGTCTTCT 3' 5' GCACATTTACTTAAGCCCTGG 3' | rs11105832 |
| LR49^b^ | 7 | A | chr15:93618994-93619116 | 5' GAGACCCCAGTCTTGCGAC 3' 5' AAGTCCCCACTTTGAAGATGTC 3' | rs12903384 |

^a^ Use to designate the mononucleotide repeat and the amplicon.

^b^ For LR49 only the length of the poly-adenine tract was scored. This tract is adjacent poly-guanine tract of length seven.
