# Supplementary material for: A novel panel of short mononucleotide repeats linked to informative polymorphisms enabling effective high volume low cost discrimination between mismatch repair deficient and proficient tumours
Source: PLoS One. 2018 Aug 29;13(8):e0203052. doi: 10.1371/journal.pone.0203052 (PMC6114912; doi:10.1371/journal.pone.0203052)
Supplement: S1 File — (GZ) [file pone.0203052.s004.gz › Redford_et_al_scripts/Content.pdf]

Directory Contents Structure

Cluster:

- MSI.test.script.sh

  Subdirectories:
- bwa.out
- fastq
- logs
- r.data
  - examplePositions.csv
- r.scripts
  - nomibinf
    - ForLinux201409021.r
- Rcm
  - exploreMNR3.Rcm
- Results

PC:

- data
  - example.test
  - .r (Result files corresponding to exampleTestSamples.csv)
  - example.train
  - .r (Result files corresponding to exampleTrainSamples.csv)
  - examplePositions.csv
  - exampleTestSamples.csv
  - exampleTrainSamples.csv
- dump
- scripts
  - combinedClassification.2.r
  - combinedCrossClassification.2.r
  - combinedScoring.r
  - extractMNR.r
  - mergeFreqBiasData.r
  - separationAnalysis.2.r
  - tabulateBias.r
  - tabulateFreqs.r
  - tabulatePointMuts.r
  - exampleMSIclassifier.r
