# Supplementary material for: A novel panel of short mononucleotide repeats linked to informative polymorphisms enabling effective high volume low cost discrimination between mismatch repair deficient and proficient tumours
Source: PLoS One. 2018 Aug 29;13(8):e0203052. doi: 10.1371/journal.pone.0203052 (PMC6114912; doi:10.1371/journal.pone.0203052)
Supplement: S1 File — (GZ) [file pone.0203052.s004.gz › Redford_et_al_scripts/Scripts.pdf]

Scripts used in the analysis

The folder contains the scripts used in the analysis
The first step of the analysis runs on a Linux cluster while the subsequent steps can be performed on a PC.

First step a (LINUX cluster running SGE)

Shell script: MSI.test.script.sh

Requires: SGE, with BWA (version 0.7.15) and R (version 3.0.0 or higher) accessible as modules
The subdirectories: Rcm, and r.scripts, where the late contains a subdirectory nomibinf.
The following files should be present: Rcm/exploreMNR3.Rcm and r.scripts/nomibinf/ForLinux201409021.r. No additional R-libraries needed.

The shell script requires editing of the following parameters:
Samples : An array containing the sample identifiers matching the file names of the fastq files
WorkingDirectoryName: A string with the name of the directory containing the fastq files
FastqRootDir: The name of the directory that contains the directory with the working directory
SamRootDir: The name of the directory where the sam files will be written
ResultsRootDir: The name of the directory where the results will be written
RLogsDir: The name of a directory where the logs will be written
Reference: The name of the reference file (indexed)
PositionsFileName: The name of a csv file containing the positions of
    The amplicon boundaries
    The boundaries of the mononucleotide repeat
    The SNP
    (See r.data/examplePositions.csv for an example)
The output is a series of intermediate files in the directory specified by the variable (ResultsRootDir).

On a PC running R 3.0.0 or higher

The scripts

Scriptfiles:

============
The scripts should be in subditectory “scripts”. They include the following files:
  scripts//extractMNR.r
  scripts//tabulateBias.r
  scripts//tabulateFreqs.r
  scripts//tabulatePointMuts.r
  scripts//mergeFreqBiasData.r
  scripts//combinedScoring.r
  scripts//combinedClassification.r
  scripts//combinedCrossClassification.r
  scripts//separationAnalysis.2.r

The scripts are loaded during the execution of the function separationAnalysis and require the R-library reshape2.

The function separationAnalysis is defined in the file scripts//separationAnalysis.2.r.
This function has the following input parameters:

- Train.raw.data: The .list containing the data for the training set
- Test.raw.data: The list containing the data for the test set

These lists have the following structure
Train.list <- list(DataDi r = "..//data", TypingDi r= "example.train",MarkerDataFile = "examplePositions.csv", SampleDataFile =" exampleTrainSamples.csv")
where

- DataDir: root directory where data files are stored
- TypingDir: within DataDir, stores .r Result files that summarise variants called from the Cluster pipeline
- MarkerDataFile: within DataDir, a .csv file containing marker data
- SampleDataFile: within DataDir, a .csv file containing sample data

Example of a call:
The directory “PC” should be set as the working directory:
The file “exampleMSIclassifier.r” contains an example of the required commands required.
source("scripts//separationAnalysis.2.r")
